# Modular endoprosthetic replacement for metastatic tumours of the proximal femur

**DOI:** 10.1186/1749-799X-3-50

**Published:** 2008-11-04

**Authors:** Coonoor R Chandrasekar, Robert J Grimer, Simon R Carter, Roger M Tillman, Adesegun T Abudu

**Affiliations:** 1 Royal Orthopaedic Hospital, Birmingham, UK

## Abstract

**Background and aims:**

Endoprosthetic replacements of the proximal femur are commonly required to treat destructive metastases with either impending or actual pathological fractures at this site. Modular prostheses provide an off the shelf availability and can be adapted to most reconstructive situations for proximal femoral replacements. The aim of this study was to assess the clinical and functional outcomes following modular tumour prosthesis reconstruction of the proximal femur in 100 consecutive patients with metastatic tumours and to compare them with the published results of patients with modular and custom made endoprosthetic replacements.

**Methods:**

100 consecutive patients who underwent modular tumour prosthetic reconstruction of the proximal femur for metastases using the METS system from 2001 to 2007 were studied. The patient, tumour and treatment factors in relation to overall survival, local control, implant survival and complications were analysed. Functional scores were obtained from surviving patients.

**Results and conclusion:**

There were 45 male and 55 female patients. The mean age was 60.2 years. The indications were metastases. Seventy five patients presented with pathological fracture or with failed fixation and 25 patients were at a high risk of developing a fracture. The mean follow up was 15.9 months [range 0–77]. Three patients died within 2 weeks following surgery. 69 patients have died and 31 are alive. Of the 69 patients who were dead 68 did not need revision surgery indicating that the implant provided single definitive treatment which outlived the patient. There were three dislocations (2/5 with THR and 1/95 with unipolar femoral heads). 6 patients had deep infections. The estimated five year implant survival (Kaplan-Meier analysis) was 83.1% with revision as end point. The mean TESS score was 64% (54%–82%).

We conclude that METS modular tumour prosthesis for proximal femur provides versatility; low implant related complications and acceptable function lasting the lifetime of the patients with metastatic tumours of the proximal femur.

## Introduction

Metastatic bone tumours commonly arise from carcinoma of the breast, bronchus, kidney, prostate and thyroid. There are many publications on bony metastases, surgical management, complications and outcomes [1-26]. The proximal femur is the commonest long bone to be affected by secondary malignant bone tumours [1,2] The use of endoprostheses for the treatment of malignant tumours of the proximal femur is well recognized and its use in metastatic disease is also becoming more common. The original endoprostheses were custom made hence there was a time delay to manufacture the implant, as a result modular implants have become popular in many centres. They have the advantage of allowing surgical treatment without delay for many tumours and they are especially useful for patients with pathological fractures due to metastases. Many pathological fractures of the proximal femur will not heal, either because of the disease process itself or because of the use of radiotherapy and in patients with good life expectancy and destruction of the upper femur, an endoprosthetic replacement is both functionally and oncologically a sensible option.

The main principle in treating any pathological fracture due to metastatic bone disease is that the fracture should be fixed in such a way that the patient can, if possible, resume as near normal function as soon as possible and that whatever fixation device is used, it should outlive the patient. The advantage of an endoprosthetic replacement over internal fixation of the proximal femur is that it will allow removal of the tumour involved area and replacement, thus minimising the risk of further tumour related problems like non-union and tumour progression [2,3]. The main potential complications of the use of endoprostheses are local recurrence, infection, aseptic loosening, mechanical failure and fracture (prosthetic or bone) [4-6]. There are many publications on the use of custom and modular proximal femoral endoprosthetic replacements [7-11]. We have used custom made endoprosthetic replacements for tumours of the proximal femur since 1970 [12] and we have been using the modular proximal femoral endoprosthetic replacements [METS prosthesis system designed by Stanmore Implants Worldwide] since they became available in 2001.

The aim of this study was to assess the clinical and functional outcomes following modular tumour prosthesis reconstruction of the proximal femur in 100 consecutive patients with metastatic tumours and to compare them with the published results of patients with modular and custom made endoprosthetic replacements.

## Patients and methods

Between 2001 to 2007, 100 consecutive patients underwent resection of the proximal femur and modular endoprosthetic replacement for metastatic disease of the proximal femur. Seventy five patients presented with pathological fracture or with failed fixation and 25 patients were at a high risk of developing fracture. The patients who were referred with metastatic tumours of the proximal femur were discussed at the multi-disciplinary meeting and the treatment option was chosen. Endoprosthetic replacement was preferred when there was a) failed fixation, b) gross destruction of the proximal femur not suitable for internal fixation c) metastatic disease with good prognosis – e.g. solitary renal metastasis. The operations were all carried out at a single institution by the oncological surgical team. Patient, tumour, treatment and outcome data on all cases was prospectively entered into a database.

All of the modular prostheses were designed and manufactured at the Department of Biomedical Engineering of the Institute of Orthopaedics of University College, London (now known as Stanmore Implants Worldwide, SIW). This modular system provides a choice of different femoral head sizes, trochanteric reattachment, femoral stem and shaft size and length. There is also an option to use a polished or hydroxyapatite coated collar at the bone-prosthesis junction in the expectation that there will be osseointegration with the prosthesis which will hopefully decrease the problem of late aseptic loosening.

All operations were performed in a clean air theatre. Antibiotic prophylaxis was given at the time of surgery and for up to 24 hours post-operatively. The tumour resection was carried out following oncological principles. In patients with secondary bone tumours with pathological fractures and failed implants with possible involvement of the hip joint a palliative reconstruction (marginal or planned involved margins) was carried out. An en bloc resection was carried out in patients without pathological fractures aiming to achieve a wide margin. Surgery was performed in the lateral position with a longitudinal incision including excision of the biopsy tract. The appropriate segment of the proximal femur was resected. In patients requiring proximal femoral replacement and whose disease spared the greater trochanter, this was osteotomised and reattached to the endoprostheses using the trochanteric reattachment plate and screws or cable-grip wires. If it was not possible to preserve the greater trochanter the abductor mechanism was sutured to vastus lateralis and fascia lata. There was also an option to reattach the abductors to the trochanteric holes in the implant with non-absorbable sutures. Trial components were used to select the appropriate size of components needed to restore limb length and stability. The femoral head was replaced with either a monopolar head or with an acetabular replacement depending on the status of the acetabulum. The hip capsule was preserved whenever possible. Prior to the reduction of the prosthesis a strong absorbable suture was used circumferentially around the capsule. The prosthesis was reduced and the capsule was tightened and repaired in a 'purse string' fashion increasing the stability. Monopolar heads were preferentially used for the reconstruction, whilst a cemented acetabular component was used in patients with either degenerative changes at the hip or with possible tumour involvement of the acetabulum. A smooth round or oval collar was used at the prosthesis-femur interface for the patients as the anticipated life expectancy was less than five years and Hydroxyapatite collars were selectively used. The stems were cemented, using low viscosity antibiotic containing bone cement, introduced with a cement gun.

We have used large monopolar heads in 95% of the patients with metastatic disease. Five patients had a cemented acetabular polyethylene cup with a 28 mm metal head. The mean length of femoral resection was 9 cm (range 4.5 cm to 21 cm) and all patients had a cemented intramedullary stem.

Following surgery patients were mobilized supervised weight bearing helped by experienced physiotherapists, progressing to full weight bearing by time of discharge at two weeks. The resection histology was reviewed at the multi disciplinary meeting and further treatment including radiotherapy was planned. At six weeks post surgery patients returned to the hospital for a period of intensive inpatient physiotherapy. Patients were followed up with three monthly appointments for two years, followed by six monthly appointments until five years post surgery.

The radiographs of patients who were alive for more than 24 months were analysed using the ISOLS guidelines [13]. Functional assessment of the surviving patients was assessed using the TESS questionnaire, a well validated patient completed assessment of function [14].

We analysed the patient and prosthetic survival, the risk of revision of the prosthesis, the incidence of failure of limb salvage because of amputation and complications like dislocation and infection following the use of the modular prosthetic replacement of the proximal femur. We have used Kaplan Meier survival curves to assess the failure rates of the prostheses. We have compared these outcomes with the published results of custom and modular proximal femoral endoprosthetic replacements. Throughout the time period of this study our unit carried out limb salvage in 99% of patients with metastatic tumours of the proximal femur using the modular system.

## Results

**Figure 1:**
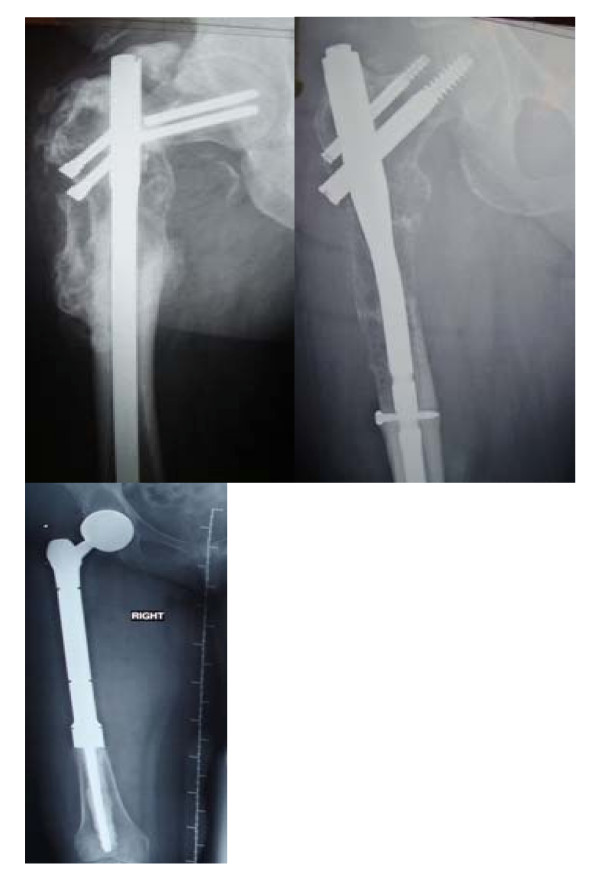
**Typical indications for a proximal femoral replacement.** a) Failed fixation of a proximal femoral fracture due to metastatic breast carcinoma b) Progressive destruction of proximal femur by metastatic renal carcinoma c) Radiograph of the modular endoprosthetic replacement at 12 months.

Between 2001 and 2007, 100 patients underwent modular endoprosthetic replacement of the proximal femur. There were 45 male and 55 female patients. The mean age was 60.2 years. The indications were metastases. The indications are shown in Table 1. Seventy five patients presented either with a pathological fracture (56 patients) or with a failed fixation (19 patients) (Figure 1a, b) and 25 patients were at a high risk of developing fracture.

**Table 1:** Patient diagnoses

Total	100
Diagnosis	Ca breast 28
	Ca renal 23
	Ca bronchus 11
	Ca prostate 5
	Ca Thyroid 3
	Adenocarcinoma 9
	Other diagnoses 21

The complications were dislocation 3%, infection 6%, local recurrence 4% and peri-operative mortality 3%.

There were three post operative dislocations of the hip. Two dislocations occurred in two of the five patients who had acetabular reconstruction using a 28 mm femoral head and a polyethylene liner (40%). One dislocation occurred in one of the 95 patients with unipolar heads (1%). The three patients had closed reduction of the dislocation. There were no early cup revisions for dislocation.

Six patients developed a wound infection of whom five were early (within three months of surgery) and one late (after three months). Three patients were treated successfully with wound debridement and antibiotics, two patients had persistent chronic infection treated with long term antibiotics and one patient eventually required a hip disarticulation. Sixteen patients were noted to have received post operative radiotherapy in the present series and none of them developed a deep infection.

Local recurrence arose in four patients (4%) after 9, 9, 12 and 25 months after the index surgery. Local recurrence occurred in patients with a previous pathological fracture. Two of these patients had palliative treatment because of widespread disease; two patients had more extensive reconstruction (one conversion to a total femur replacement and Harrington reconstruction of the acetabulum).

One patient had a hip disarticulation for infection. The limb salvage rate was 99% for the present series.

**Figure 2:**
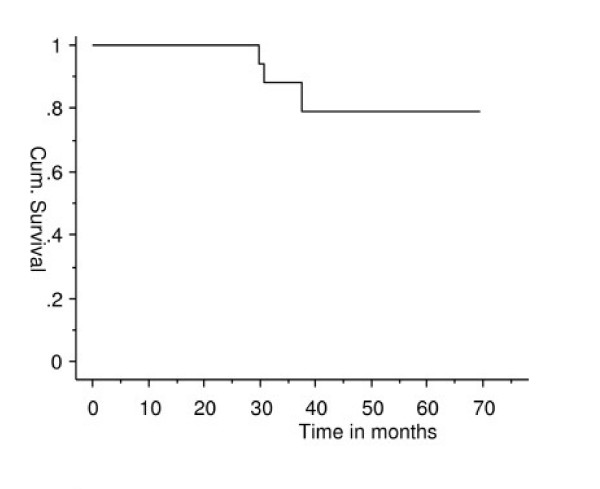
Prosthetic survival without revision.

Three patients required revision surgery. (Two for local recurrence and acetabular erosion, one had revision to total femur for tumour progression). We have used large monopolar heads in 95% of the patients with metastatic disease and only 2% (2 patients) needed further revision surgery for acetabular erosion. The estimated one and five year implant survival was 100% and 83.1% with revision as end point (Figure 2).

**Figure 3:**
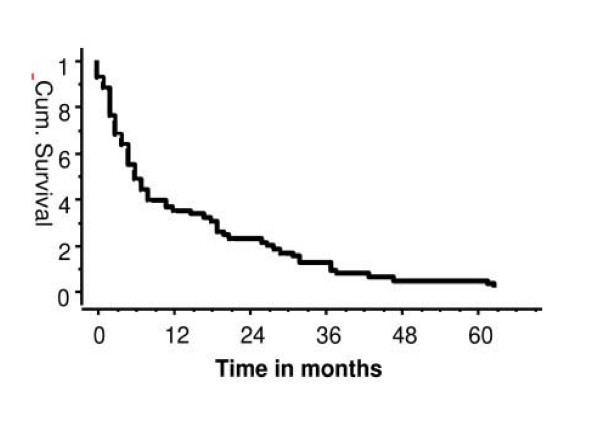
Patient survival after surgery (35% at 1 year, 21% at 2 years and 10% at 3 years).

**Figure 4:**
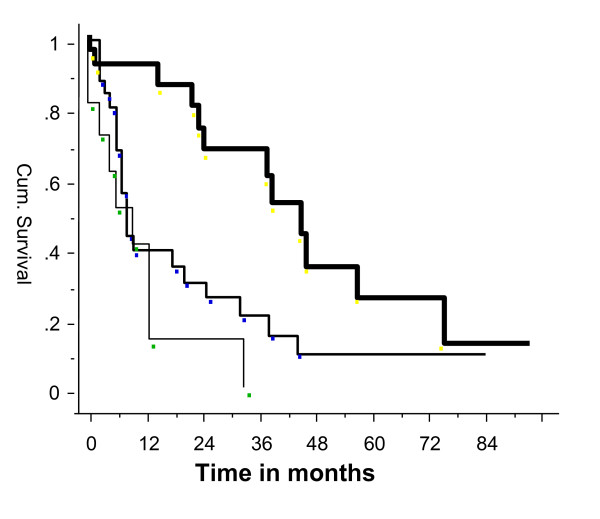
Patient survival after endoprosthetic replacement of the proximal femur for metastatic renal, breast and bronchogenic carcinoma (renal – right, middle – breast and left – bronchus).

The mean follow up was 15.9 months (range 0–77 months). There were three perioperative deaths, due to pulmonary embolism in elderly patients who had been on prolonged bed rest prior to the operation, and a further three patients had a pulmonary embolism postoperatively. Sixty nine patients have died and 31 are alive. Of the 69 patients who were dead 68 did not need revision surgery indicating that the implant provided single definitive treatment which outlived the patient. One patient had revision surgery. The estimated one, two and three year patient survival (Kaplan-Meier analysis) was 35%, 21% and 10% respectively (Figure 3). Twenty five patients lived more than two years after the surgery. Eleven of these patients had metastatic renal carcinoma and six had metastatic breast carcinoma. The estimated one year patient survival (Kaplan-Meier analysis) after the proximal femoral endoprosthetic replacement for metastatic renal, breast and bronchogenic carcinoma was 86%, 40% and 10% respectively (Figure 4).

The radiographs of 25 patients who survived more than 24 months were analysed according to the ISOLS guidelines [13]. Greater trochanter related problems were seen in six patients who had sparing and reattachment (3 proximal migration, 2 broken wires and one calcification). No other patient had any adverse features on the radiographs.

A functional assessment questionnaire (the TESS score) was sent to all surviving patients. The mean TESS score for patients with metastatic bone tumours was 64% (54% – 82%).

## Discussion

Limb salvage using proximal femoral endoprosthetic replacements, allografts and allograft prosthesis composite have been reported [4,7,8,10-12]. Long term results of custom proximal femoral replacement showed implant survival without revision was 77% at 10 years and 57% at 20 years [12]. Custom implants are not readily available and for patients with pathological fractures and failed trauma implant fixations of the proximal femur, custom implants are not ideal due to the delay in their availability, resulting in enforced bed rest with the associated morbidity. Occasionally, tumour progression during this delay may compromise the margins of resection if a custom made prosthesis is chosen. Based on the extensive experience in the use of custom endoprostheses, Stanmore Implants Worldwide introduced modular endoprosthetic replacement of the proximal femur in 2001.

One hundred patients with metastatic bone tumours formed the present series and most of these patients had an actual or impending pathological fracture or a failed implant. Primary bone tumours formed the major indication for proximal femoral replacement in most of the published series [4,7,11,12]. The success of a prosthesis is judged by its ability to provide a solid, functioning prosthesis without complications for the remainder of that patient's life. This has been achieved for 68 of the 69 patients (98.5%) who died with the implant in situ, without any revision being required.

The problem of infection following proximal femoral endoprosthetic replacement has been highlighted by several authors [4,6,11,12]. The rate of reported infection varied from 1.2% to 19.5%.(Table 2). The rate of infection in the present series was 6% and this is comparable to the incidence of 6.3% reported by Menendez et al [7] using a modular prosthesis in a series of 96 patients but is considerably lower than the rate of infection of 19.5% reported by Gosheger et al [11] in 41 patients who underwent modular proximal femoral endoprosthetic replacement. This high infection rate was attributed to 4 patients receiving post operative radiotherapy following resection of Ewing's sarcoma. Radiotherapy is known to be a significant risk factor in relation to infection of the endoprosthetic replacements [6]. In our series 16 patients received radiotherapy and none of them developed deep infection.

**Table 2:** Comparing complications and implant survival of the published series of custom and modular proximal femoral endoprosthetic replacement with the present series

Author	Infection	Local Recurrence	Dislocation	Revision	5 Year Survival
Kabukckuoglu^12 ^[custom] 54 patients [1972–1992]	1.8%	28%	11%	17%	
Unwin^4 ^[custom] 263 patients [1968–92]	2.7%*	4.6%*		6.1%	
Gosheger^11 ^[modular] 41 patients [1992–03]	19.5%		7.3%		78.7%
Menendez^7 ^[modular] 96 patients [1992–03]	6.3%	3.1%	10.4%	7.3%	82%
Present Series [modular] 100 patients [2001–07]	6%	4%	3%	3%	83.1%

* had revision surgery

Dislocation is a well recognized complication with proximal femoral endoprosthetic replacement with the reported rates of dislocation varying from 1.7% to 20% [7,11,12]. This is due to the extensive resection of soft tissues around the hip, including muscles and hip capsule in most cases. Repairing both the hip capsule and the abductor lever arm is difficult. Most authors have reported a high dislocation rate with the use of small femoral head sizes in this location after tumour resection and larger head sizes do seem preferable to try and reduce this. The dislocation rate of 3% in the present series is comparable to other reported series (Table 2). The dislocation rate was 17% in a series of 54 patients with primary bone tumours treated with custom implants from our centre [12]. The use of a monopolar large femoral head resulted in the dislocation rate being reduced to 3% in the present series. Two of the five patients who had a total hip type of reconstruction had a dislocation. We have used large monopolar heads in 95 patients with metastatic disease and only 2% (2 of the 95) needed further revision surgery for acetabular erosion indicating that large monopolar heads can be safely used for patients with metastatic disease without acetabular involvement. We used monopolar heads in this series specifically to reduce the risk of dislocation.

Aseptic loosening is a well recognised complication with the use of custom and modular implants with long term follow up [4,11,12]. We have used hydroxyapatite coated collars for patients with anticipated long term survival to reduce the risk of aseptic loosening especially in patients with metastatic renal carcinoma. This has been shown to be very effective for both distal femoral and proximal tibial replacements [16,17]. Long term follow up will be needed to assess whether this is equally effective for proximal femoral replacements.

Pathological fractures of the proximal femur may not heal despite internal fixation with intramedullary nail and post operative radiotherapy. This is more common with metastatic renal carcinoma [15,18,23,26]. The tumour can progress causing the eventual failure of the implant necessitating further surgery. The risk of reoperation following failed internal fixation for metastases is between 20% – 35% [18,19,25]. This is related to the type of the metastatic tumour and duration of survival. The present study has shown that 86% of the patients with metastatic renal carcinoma and 40% of the patients with metastatic breast carcinoma were alive at one year following the endoprosthetic replacement surgery. The long term survival of patients with metastatic renal carcinoma is well known [15,23,26]. The local failure rate following internal fixation was 24% for metastatic renal carcinoma [23]. Hence primary endoprosthetic replacement should be considered as a treatment option for patients with renal metastases as the failure rate of the endoprostheses is low compared with internal fixation. Wedin et al [15] recorded a 14% failure rate for osteosynthetic devices compared with 2% for the endoprosthesis. Because of the low failure rate the endoprosthesis is more cost effective and it provides a strong, permanent, stable construct that allows immediate return to functional mobility lasting the lifetime of the patient with the metastatic disease of the proximal femur.

The justification for using proximal femoral replacement surgery with a one year mortality of 65% is debatable. Wedin et al [18] reported 30% one year, 10% two years and 7% three years patient survival following surgery for proximal femoral metastases. The estimated one, two and three year patient survival for the present series is 35%, 20% and 10% respectively. The estimated one year survival for patients with metastatic renal carcinoma was 86%. For a patient with metastatic disease of the proximal femur often with pathological fracture and failed fixation, surgery with its inherent risks and the potential benefits is often the better option than bed rest, palliation and radiotherapy. Patients with a pathological fracture not suitable for internal fixation or failed fixation with a life expectancy of at least six weeks were offered the option of proximal femoral endoprosthetic replacement An informed choice was made by the patient based on the recommendations of the multi disciplinary team. The patients who died within the first year had a stable pain free proximal femur. This enabled them to have some mobility and dignity during the precious last few months of their lives improving the quality of life.

The overall risk of amputation following modular proximal femoral replacement in this series was 1% and was directly related to the risk of infection. We conclude that in our hands a modular proximal femoral endoprosthesis has fulfilled its aim of providing reasonable function with a low rate of complications improving the quality of life for the patients with metastatic disease of the proximal femur. We recommend the use of proximal femoral endoprosthetic replacement for patients with proximal femoral metastases with a) failed fixation, b) gross destruction of the proximal femur not suitable for internal fixation c) metastatic disease with good prognosis. A monopolar head can be safely used for most patients and if there is acetabular involvement or degeneration a cemented acetabular replacement is indicated.

## Authors contributions

All authors contributed to the article.  

## Competing interests

The authors declare that they have no competing interests.
